# Albumin Deficiency Reduces Hepatic Steatosis and Improves Glucose Metabolism in a Mouse Model of Diet-Induced Obesity

**DOI:** 10.3390/nu15092060

**Published:** 2023-04-25

**Authors:** Afsoun Abdollahi, Sanjeev K. Narayanan, Alexandra Frankovich, Yen-Chun Lai, Yi Zhang, Gregory C. Henderson

**Affiliations:** 1 Department of Nutrition Science, Purdue University, West Lafayette, IN 47907, USA; 2 Department of Comparative Pathobiology, Purdue University, West Lafayette, IN 47907, USA; 3 Division of Pulmonary, Critical Care, Sleep and Occupational Medicine, Indiana University School of Medicine, Indianapolis, IN 46202, USA; 4 Department of Anatomy, Cell Biology and Physiology, Indiana University School of Medicine, Indianapolis, IN 46202, USA

**Keywords:** non-esterified fatty acid, fatty liver disease, diabetes, insulin resistance, analbuminemia, diet-induced obesity

## Abstract

Serum albumin facilitates the transport of free fatty acids (FFAs) from adipose tissue to other organs. It was not known if impeding this process could protect from hepatic steatosis and metabolic dysfunction in obesity. We tested whether albumin knockout (Alb^−/−^) mice would exhibit a reduction in plasma FFA concentration, reduced hepatic lipid accumulation, and improved glucoregulation as compared to wild-type (WT) mice. Male homozygous albumin knockout mice (Alb^−/−^) and WT controls were fed a low-fat diet (LFD) or high-fat diet (HFD). Alb^−/−^ mice exhibited a similar body weight gain and body composition as WT on both diets. Despite HFD-induced obesity, Alb^−/−^ mice were protected from various comorbidities. Compared to WT mice on the HFD, Alb^−/−^ exhibited lower plasma FFA levels, lower blood glucose levels during glucose tolerance and insulin tolerance tests, and lower hepatic steatosis and inflammation. Alb^−/−^ mice on HFD also exhibited elevated expression of multiple genes in the liver and adipose tissues, such as peroxisome proliferator-activated receptor α in both tissues, as well as glucose transporter-4 and adiponectin in adipose tissues. The results indicate that albumin’s FFA transport function may be involved in the development of hepatic lipid accumulation and dysregulated glucose metabolism in obesity.

## 1. Introduction

Type 2 diabetes (T2D) and non-alcoholic fatty liver disease (NAFLD) are major public health problems, and the incidence of each disorder is linked to obesity [1,2,3,4,5]. T2D afflicts approximately 10% of Americans [6] while NAFLD has an incidence of approximately 30% [7]. Hepatic steatosis is associated with numerous metabolic dysfunctions including obesity, metabolic syndrome, T2D, and other cardiometabolic abnormalities [2,8]. To reflect the pathogenesis of NAFLD and its intimate association with dysregulated metabolism, the condition has more recently been referred to as metabolic (dysfunction)-associated fatty liver disease (MAFLD) [9]. Because liver health impacts glucoregulation, preventing NAFLD development could reduce insulin resistance and T2D risk.

In hepatic steatosis, triacylglycerols (TAGs), cholesterol esters (CEs), and lipotoxic intermediates cause cellular dysfunction [2]. Obesity can also lead to ectopic lipid deposition in cardiac muscle and other tissues [10]. Plasma FFAs predominantly arise from lipolysis in adipose tissues and are exported via FFA transporters. However, the final step in the FFA export process is binding to albumin which facilitates the dissolution of hydrophobic FFAs into the plasma, and it is important to consider albumin’s role in FFA trafficking. While albumin has multiple functions such as binding hormones and different metabolites, FFAs are considered to be albumin’s primary ligand [11,12,13] and thus albumin is centrally involved in lipid metabolism. Hepatic steatosis and the associated lipotoxicity are associated with impaired insulin sensitivity [4,14,15,16]. Hepatic ceramide and diacylglycerol (DAG) accumulation are each considered potential culprits in insulin resistance related to steatosis [14,17,18,19,20], and steatosis-related inflammation also appears to be linked to insulin sensitivity [21]. Therefore, it may be metabolically beneficial to sequester lipids in adipose tissues, particularly if the adipose tissues’ metabolic regulation can adapt to this metabolic challenge. 

Interventions to reduce plasma FFA concentrations could be expected to improve health [22]. There is a need to identify metabolic processes that control the plasma FFA concentration and FFA transport between tissues. Over the years, it has been a goal to discover therapeutic approaches for reducing plasma FFA concentrations, with the focus generally on lipolysis. Nicotinic acid treatment was potentially promising because it can prevent lipolysis; however, it has intolerable side effects [23] and the efficacy is not maintained over long-term treatment [24,25]. Adipose triglyceride lipase (ATGL) is a rate-limiting enzyme for the control of lipolysis, and thus controlling this enzyme’s activity could be another potential avenue for modulating plasma FFAs. Whole-body knockout (KO) of ATGL improved insulin sensitivity but also caused severe steatosis in the heart and liver, as well as cardiac dysfunction [26]. Thus, blocking this rate-limiting lipolytic enzyme in the entire body may not be a viable modality for improving metabolic health. When testing other approaches for reducing plasma FFA levels, it would be important to confirm that the intervention or gene KO does not cause steatosis in the heart or liver. Adipose tissue-specific KO of ATGL decreased plasma FFA levels and improved insulin sensitivity without worsening hepatic or cardiac steatosis [27]. However, this tissue-specific KO model exhibited exacerbated weight gain. To date, there is no well-accepted therapeutic target for reducing plasma FFA concentrations as a means of preventing ectopic lipid deposition and improving insulin sensitivity. Therefore, research efforts continue in an effort to develop a deeper understanding of potential approaches to improve metabolic health through alterations in plasma FFA trafficking. 

As FFA molecules are hydrophobic and depend upon binding to albumin for dissolution/transport in the blood, our efforts to understand FFA metabolism in the context of insulin resistance and hepatic steatosis are focused on studying albumin. In our previous report, we discovered that Alb^−/−^ mice on a chow diet exhibited lower plasma FFA, hepatic lipid, and blood glucose concentrations than wild-type (WT) mice during oral glucose tolerance testing (OGTT) and insulin tolerance testing (ITT), and no abnormal fatigue levels [28]. However, it was not known if this superior metabolic health would be exhibited under obesogenic conditions when dietary fat intake was high and insulin resistance was induced by diet. 

We sought to study the metabolic response of albumin KO (Alb^−/−^) mice to a high-fat diet (HFD) as compared to a low-fat diet (LFD). We tested the role of albumin in determining plasma FFA concentrations, its effects on lipid metabolism in the liver and adipose tissues, and its impacts on glucose metabolism, substrate oxidation, and body composition. We tested for the potential metabolic benefits of reducing plasma FFA concentrations in obesity by targeting albumin. The results provide a characterization of the role of albumin in the processes that lead to metabolic dysregulation and insulin resistance in obesity. 

## 2. Materials and Methods

Mouse model. This protocol was approved by the Purdue University Animal Care and Use Committee. All mice in this study were males on the C57BL/6J genetic background. WT mice (strain #000664) and Alb^−/−^ mice (strain #025200) were purchased from the Jackson Laboratory (Bar Harbor, ME). Alb^−/−^ is a whole-body KO model [29]. As albumin is normally expressed only in the liver, the impact of gene ablation was on the liver. 

Experiment 1. WT and Alb^−/−^ mice, fed either an LFD or HFD (*n* = 6 per group), were housed in an animal facility at Purdue University and maintained on a 12 h light/dark photoperiod. The mice underwent in vivo phenotyping assessments and analyses of tissues. The mice were allowed ad libitum access to food and water except for a brief withdrawal of food before specific procedures as described below. All mice consumed the Labdiet 5K52 diet (Purina Mills, St. Louis, MO, USA) until 10 weeks of age. The LFD (D12450J) and HFD (D12492) from Research Diets (New Brunswick, NJ, USA) were introduced at 10 weeks of age and were fed for eight weeks. The LFD (control diet) consists of 10% energy from fat, and the HFD (obesogenic diet) consists of 60% energy from fat. One may reasonably consider a diet with 30% or less energy from fat as one that is low in fat and greater than 35% energy as one that is high in fat. As is typical in mouse model research, the LFD in the present study had a fat content that was significantly below this threshold for a diet low in fat and the HFD was significantly beyond the threshold for a diet that is high in fat. Mice were euthanized at 18 weeks of age following a 4–6 h fast. Food intake was measured by weighing food weekly.

Experiment 2. To confirm the presence of robust plasma FFA suppression in Alb^−/−^ mice, an additional study with a longer fast (16 h) was performed. For the analysis of FFAs, plasma was collected from mice with the same genotypes, diet feeding, and ages described for Experiment 1, but with a 16 h fast before euthanasia. 

Indirect calorimetry. At 16 weeks of age, oxygen consumption (VO_2_) and carbon dioxide production (VCO_2_) of mice allowed free access to food and water were measured over a 24 h period with an Oxymax System (Columbus Instruments, Columbus, OH, USA). The respiratory quotient (RQ) was calculated as VCO_2_/VO_2_, and standard equations were used to calculate energy expenditure. 

Oral glucose tolerance test. An OGTT was performed at 17 weeks of age, following a 4–6 h fast. Glucose (2 g/kg body weight) was administered by orogastric gavage as a 20% wt/vol solution. Blood glucose levels were measured in tail blood using a glucometer (Prodigy Diabetes Care, Charlotte, NC, USA) at times 0, 10, 20, 30, 60, 90, and 120 min. The area under the curve (AUC) was calculated as a Riemann Sum (i.e., the trapezoidal rule).

Insulin tolerance test. ITT was performed at 17 weeks of age, following a 4–6 h fast. Insulin (0.75 U per kg body weight) was administered by intraperitoneal injection. Blood glucose levels were measured and the data were analyzed as described above for the OGTT. 

Exercise performance test. To determine if albumin deficiency led to an altered ability to exercise, at 18 weeks of age, time to exhaustion in an incremental treadmill exercise test was assessed as described previously [28]. The test duration at which each mouse reached exhaustion was noted.

Tissue collection. Immediately following euthanasia by CO_2_ inhalation, blood was collected by cardiac puncture, placed in ethylenediaminetetraacetic acid tubes, and centrifuged to obtain the plasma. The liver, heart, and epididymal adipose tissues were quickly collected. A portion of the liver and heart was fixed in 10% neutral buffered formalin with the remaining tissue frozen at −80 °C until analysis. 

Histology. Hematoxylin and eosin (H&E) and oil red-O (ORO) staining of sectioned liver and heart tissues were performed in the Histology Research Laboratory at Purdue University. Histological slides were examined using an Olympus BX43 microscope and assessed by veterinary pathologists. The images were captured using an Olympus DP27 camera and processed using CellSens Entry software version 1.18. For ORO staining, the slides were graded on a numeric scale for the extent of steatosis.

Biochemical assays. The FFA concentration in the plasma samples was measured using a colorimetric assay (Millipore-Sigma, St. Louis, MO, USA). The plasma samples were also analyzed in the Translation Core at Indiana University School of Medicine using automated assays for total cholesterol (TC), high-density lipoprotein cholesterol (HDL-C), TAG, alanine aminotransferase (ALT), and aspartate aminotransferase (AST); the same core laboratory also measured insulin using an enzyme-linked immunosorbent assay (Merdodia, Minneapolis, MN, USA). For measurement of TAG concentration in the liver, the tissue was homogenized and extracted with heptane, followed by the use of an assay kit as described previously [28]. For the analyses of DAGs, CEs, and ceramides, liver tissues were homogenized and extracted with chloroform and analyzed by the mass spectrometry approach as described previously [28]. For individual ceramide species analysis, those with both a fold change of 1.5 or greater, and showed a significant effect of genotype in the analysis of variance (ANOVA) results, were selected.

mRNA quantitation. Approximately 100 mg of adipose tissue and 10 mg of liver were each processed with a bead homogenizer in Trizol Reagent. RNA was isolated using kits (Qiagen, Hilden, Germany). cDNA was synthesized from RNA using the AffinityScript qPCR cDNA Synthesis Kit (Agilent Technologies, Santa Clara, CA, USA). Taqman qPCR was performed using a QuantStudio 7 Real-Time PCR System (Applied Biosystems, Waltham, MA, USA). The primers and probes are listed in Table 1. All samples were analyzed in triplicate with 18S ribosomal RNA as the control and the results were calculated using the comparative Ct method.

Western blot. For the determination of inflammation in the liver using Western blot, the samples were prepared using a procedure described previously [28]. The samples were then run on 4–15% polyacrylamide gels by electrophoresis, transferred to nitrocellulose membranes (BioRad, Hercules, CA, USA), and then blocked with 5% nonfat dried milk. The primary antibodies were against NF-κB (p65), phosphorylated NF-κB (phospho-p65), and β-actin (1:1000 dilutions; Cell Signaling Technology, Danvers, MA, USA). The secondary antibodies were conjugated to IR Dye 680 or IR Dye 800 (1:15,000 dilutions; LI-COR Biosciences, Lincoln, NE, USA). The incubation and washing steps were described previously [28], and membranes were imaged by fluorescence (LI-COR Biosciences) with values normalized to β-actin. 

Proteomics. An equal volume of each plasma sample (1 μL) was prepared for global proteomic analysis and analyzed by tandem mass spectrometry in the Purdue Proteomics Facility using label-free quantitation as described previously [28]. Proteins with greater than a single zero in a WT group were removed from the dataset. Proteins with both a fold change of 1.5 and showed a significant difference between genotypes using ANOVA were selected.

Statistical analysis. The data are presented as mean ± SE. The results were analyzed using ANOVA, with genotype and diet as the independent factors, and time as a factor when appropriate. For the analyses of VO_2_ and metabolic rate, analysis of covariance (ANCOVA) was used with body weight as the covariate. Fisher’s least significant difference post hoc test was used. Statistical analyses were performed using JMP version 16 (SAS Institute Inc., Cary, NC, USA) with *p* < 0.05 considered statistically significant.

## 3. Results

**Figure 1:**
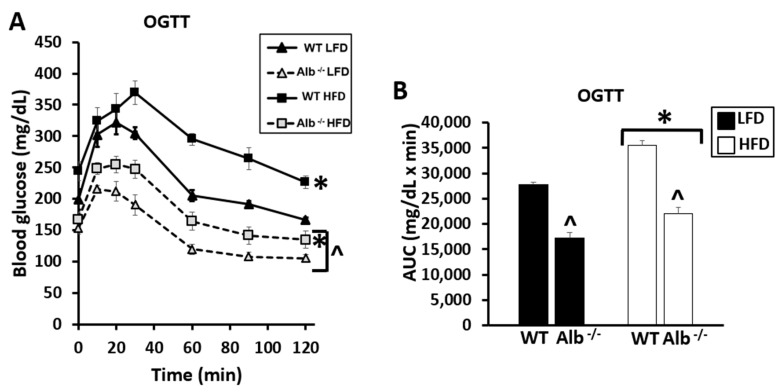

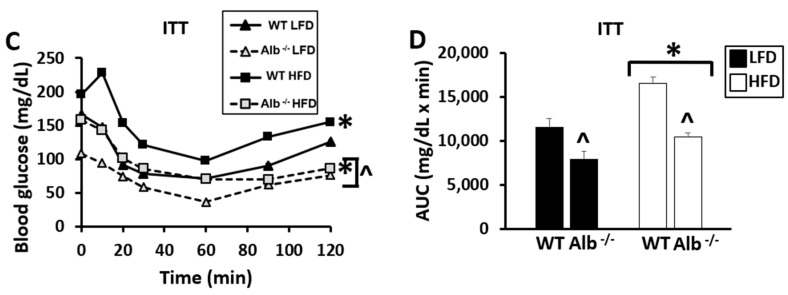
Oral glucose tolerance test (OGTT) and insulin tolerance test (ITT). Blood glucose over time for OGTT (**A**) and ITT (**C**). AUC (area under the curve) for blood glucose concentration in OGTT (**B**) and ITT (**D**). *n* = 6 mice per group. Analysis using ANOVA. ^ Alb^−/−^ significantly different from WT for each individual time point and for AUC (main effect of genotype, *p* < 0.05). * HFD significantly different from LFD for each individual time point and for AUC (main effect of diet, *p* < 0.05). LFD, low-fat diet; HFD, high-fat diet. Values are means ± S.E.

For blood glucose levels during the OGTT and ITT, Alb^−/−^ exhibited lower blood glucose levels than WT at all time points. There was a main effect of genotype (*p* < 0.05), main effect of diet (*p* < 0.05), and main effect of time (*p* < 0.05) (Figure 1A,C). The glucose AUC was also lower in Alb^−/−^ than WT (main effect of genotype, *p* < 0.05) and higher in HFD than LFD (main effect of diet, *p* < 0.05) (Figure 1B,D). These group differences in glucoregulation were present despite the observation that food intake and body weights were not significantly different between the genotypes (Table 2). There was a genotype-by-diet interaction for fasting plasma insulin concentration; insulin was lower in Alb^−/−^ than WT mice when on a HFD (*p* < 0.05) (Table 2).

**Figure 2:**
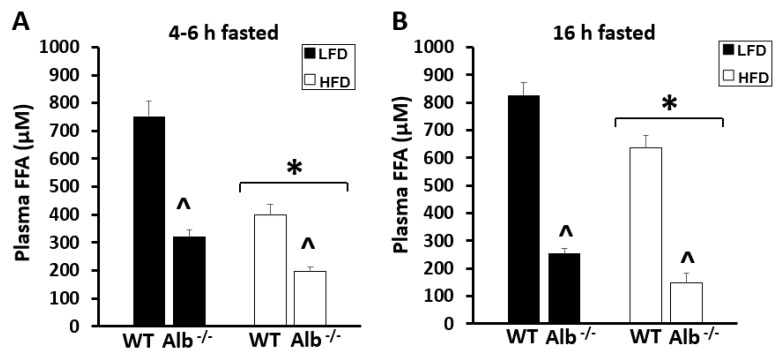
Plasma free fatty acid (FFA) concentration after 4–6 h fasting (**A**), and after 16 h fasting (**B**). *n* = 5–6 mice per group. Analysis using ANOVA. ^ Alb^−/−^ significantly different from WT within a diet (*p* < 0.05). * HFD was significantly different from LFD for both genotypes (*p* < 0.05). LFD, low-fat diet; HFD, high-fat diet. Values are means ± S.E.

After 4–6 h of fasting, Alb^−/−^ exhibited lower plasma FFA concentrations than WT (Figure 2A); there was a main effect of genotype (*p* < 0.05), main effect of diet (*p* < 0.05), and an interaction (*p* < 0.05). The post hoc test indicated that the plasma FFA concentration in Alb^−/−^ mice was lower than that of WT on each diet (*p* < 0.05). To confirm the lower plasma FFA levels in Alb^−/−^ mice vs. WT, we also tested a longer fast (16 h) (Figure 2B). There were main effects of genotype (*p* < 0.05) and of diet (*p* < 0.05), confirming the results observed with a 4–6 h fast. As we consider the 4–6 h fast to be more representative of conditions encountered in humans, all other experiments were performed with the 4–6 h fast. While the primary circulating lipid class of interest was FFAs, we were aware that other lipid fractions may respond to albumin deficiency, and therefore lipoproteins were assessed. There was a main effect of genotype for plasma TC levels (*p* < 0.05) but not for plasma TAG or HDL-C (Table 2).

**Figure 3:**
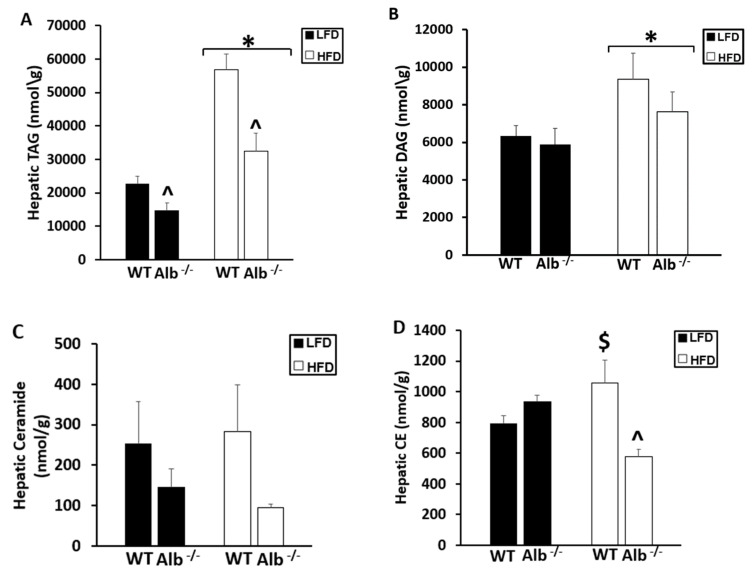
Triacylglycerol (TAG) (**A**), diacylglycerol (DAG) (**B**), ceramide (**C**), and cholesteryl ester (CE) (**D**) concentrations in the liver. *n* = 5–6 mice per group. Analysis using ANOVA. ^ Alb^−/−^ significantly different from WT within a diet, *p* < 0.05. * HFD significantly different from LFD (main effect of diet, *p* < 0.05). $ different from WT on LFD. LFD, low-fat diet; HFD, high-fat diet. Values are means ± S.E.

**Figure 4:**
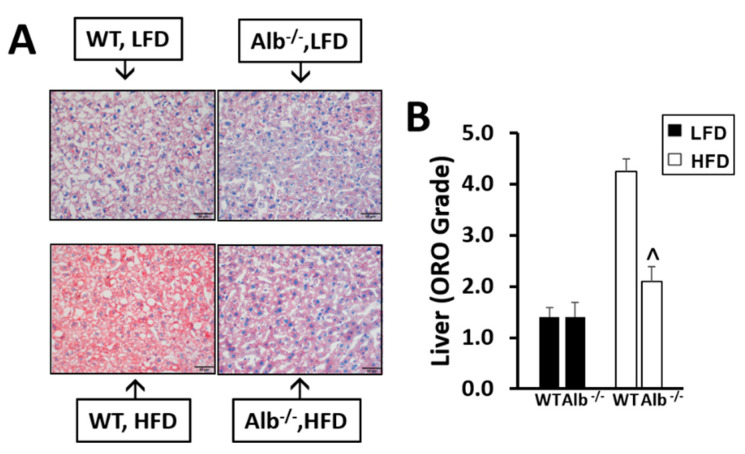

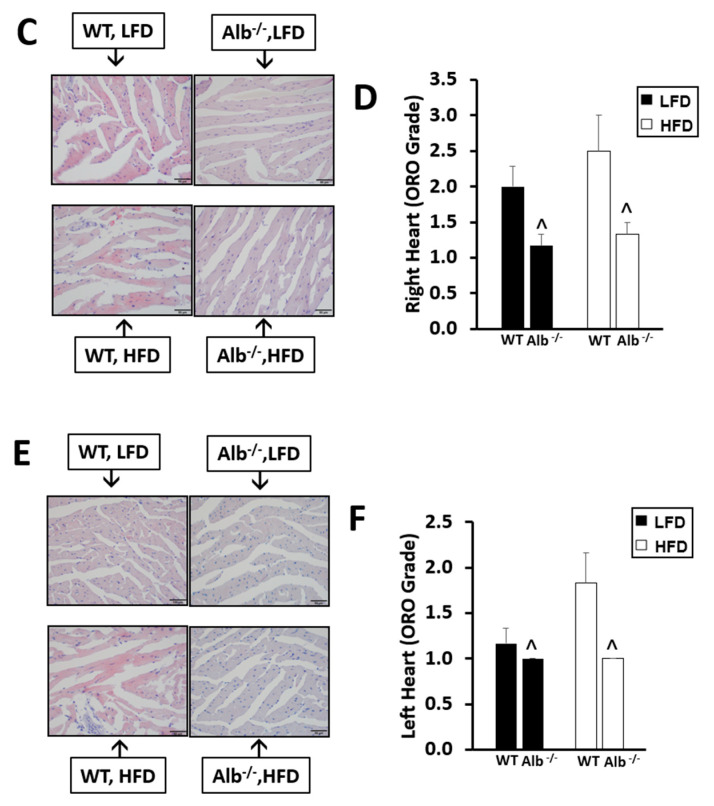
Visualization and quantification of neutral lipids via ORO analysis of liver tissues (**A**,**B**), right heart (**C**,**D**), and left heart (**E**,**F**). Scale bars, 50 μm; magnification is ×400. *n* = 4–5 per group for liver and *n* = 3 per group for heart. Analysis using ANOVA. ^ Alb^−/−^ significantly different from WT, *p* < 0.05. LFD, low-fat diet; HFD, high-fat diet. Values are means ± S.E.

Hepatic TAG concentrations were elevated by HFD (main effect of diet, *p* < 0.05) and were lower in Alb^−/−^ than WT (main effect of genotype, *p* < 0.05), with no significant diet-by-genotype interaction (Figure 3A). DAG levels were not significantly different between genotypes (Figure 3B), but ceramide concentrations tended to be lower in Alb^−/−^ than WT (*p* = 0.1 for main effect of genotype. Figure 3C). Individual ceramide species analysis revealed lower d18:1/20:0 ceramide levels in Alb^−/−^ mice compared to WT mice on both LFD and HFD (*p* < 0.05). For hepatic CEs (Figure 3D), ANOVA indicated a significant genotype-by-diet interaction (*p* < 0.05); CE levels were lower in Alb^−/−^ than WT on HFD (*p* < 0.05). ORO staining of the liver tissues (Figure 4A,B) indicated a genotype-by-diet interaction (*p* < 0.05); ORO staining was lower in Alb^−/−^ than WT when on the HFD (*p* < 0.05). To evaluate the possibility of cardiac steatosis as an untoward result of plasma FFA suppression, we also performed ORO staining on right ventricle (Figure 4C,D) and left ventricle (Figure 4E,F) tissues; Alb^−/−^ mice did not suffer from cardiac steatosis. Compared to WT in both LFD and HFD, Alb^−/−^ mice actually exhibited lower ORO staining (main effect of genotype, *p* < 0.05). H&E staining of the liver and heart did not indicate any other differences in tissue characteristics between WT and Alb^−/−^.

**Figure 5:**
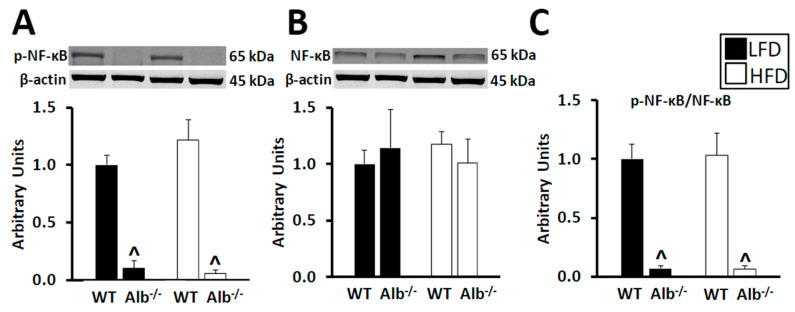
Nuclear factor kappa B (NF-κB) expression in the liver. (**A**) Western blot for phosphorylated p65 (subunit of NF-κB), (**B**) total p65, and (**C**) the ratio of phosphorylated to total p65. *n* = 5 per group. Band intensity normalized to β-actin, and the scale of the arbitrary unit was adjusted such that the average WT LFD would be a value of 1. Analysis using ANOVA. ^ Alb^−/−^ significantly different from WT, *p* < 0.05. LFD, low-fat diet; HFD, high-fat diet. Values are means ± S.E.

**Figure 6:**
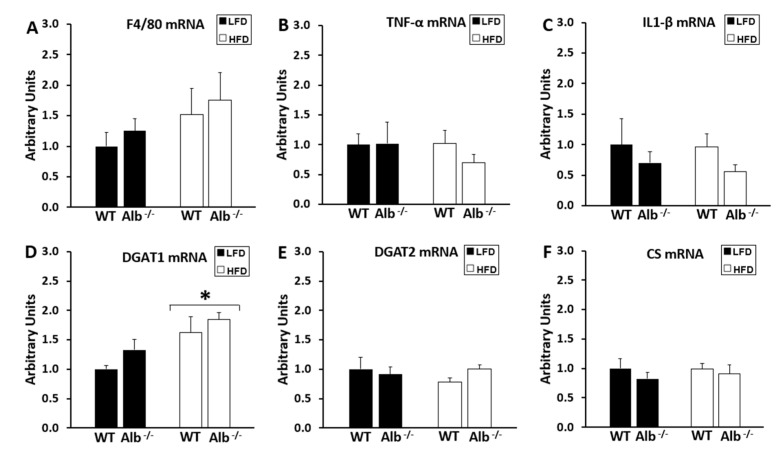

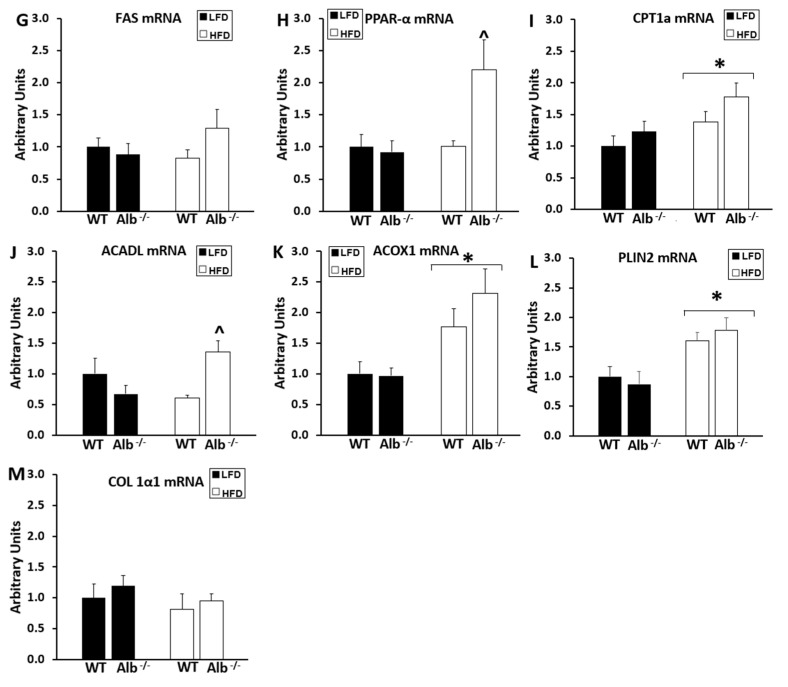
Gene expression in the liver. Gene name for the mRNA expression is shown as the title in each subfigure (**A**–**M**). *n* = 4–6 mice per group. Gene expression normalized to 18S. Analysis using ANOVA. ^ Alb^−/−^ significantly different from WT within a diet (*p* < 0.05), * LFD significantly different from HFD, *p* < 0.05. LFD, low-fat diet; HFD, high-fat diet. Values are means ± S.E.

**Figure 7:**
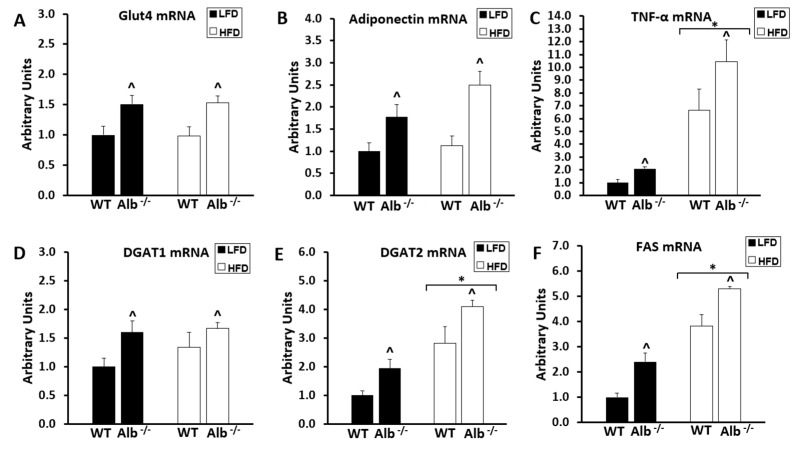

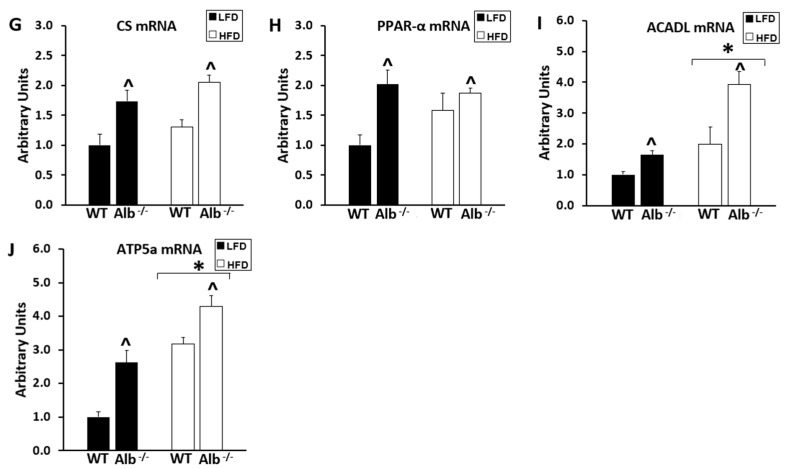
Gene expression in adipose tissues. Gene name for the mRNA expression is shown as the title in each subfigure (**A**–**J**). *n* = 4–6 mice per group. Gene expression normalized to 18S. Analysis using ANOVA. ^ Alb^−/−^ significantly different from WT (main effect of genotype, *p* < 0.05). * HFD is significantly different from LFD, *p* < 0.05. LFD, low-fat diet; HFD, high-fat diet. Values are means ± S.E.

Phosphorylated p65 protein and its ratio to total p65 protein was lower in Alb^−/−^ than WT in the liver tissue analysis (main effect of genotype, *p* < 0.05) (Figure 5A,C). Total p65 protein in the liver was not significantly different between groups (Figure 5B). Liver gene expression results (Figure 6A–M) indicated genotype-by-diet interactions (*p* < 0.05) for PPAR-α (Figure 6H) and ACADL (Figure 6J) expression, which were higher in Alb^−/−^ than WT on HFD (*p* < 0.05). There was also a trend for higher CPT1a expression in Alb^−/−^ compared to WT (main effect of genotype, *p* = 0.09). We also tested gene expression related to energy substrate metabolism in adipose tissues (Figure 7A–J). Adipose gene expression was higher in Alb^−/−^ than WT (main effect of genotype, *p* < 0.05) for Glut4, adiponectin, DGAT1, DGAT2, FAS, CS, PPAR-α, ACADL, and ATP5a. 

**Figure 8:**
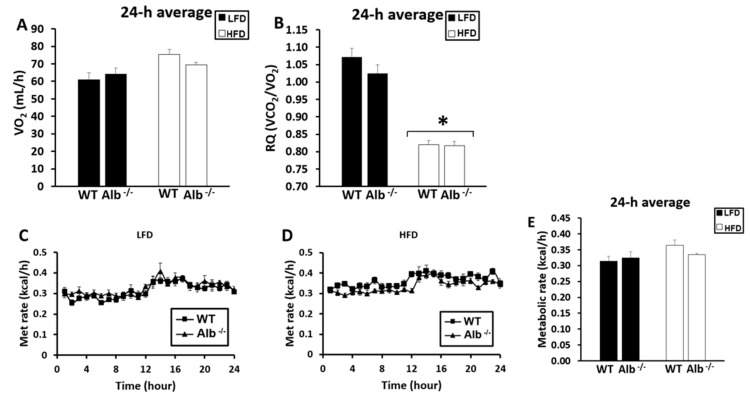
Indirect calorimetry results. Twenty-four-hour average for oxygen consumption (VO_2_) (**A**) and respiratory quotient (RQ) (**B**); metabolic rate over time (**C**,**D**) and expressed as a 24 h average (**E**). *n* = 5–6 mice per group. Analysis of RQ using ANOVA. Analysis of VO_2_ and metabolic rate using ANCOVA with body weight as the covariate. * HFD significantly different from LFD, *p* < 0.05. LFD, low-fat diet; HFD, high-fat diet. Values are means ± S.E.

In the indirect calorimetry analysis (Figure 8A–E), a main effect of diet (*p* < 0.05) was observed for RQ with HFD causing a lower RQ. There were no significant differences for metabolic rate between WT and Alb^−/−^ mice. The RQ was slightly above 1 on LFD, likely indicating de novo lipogenesis.

**Figure 9:**
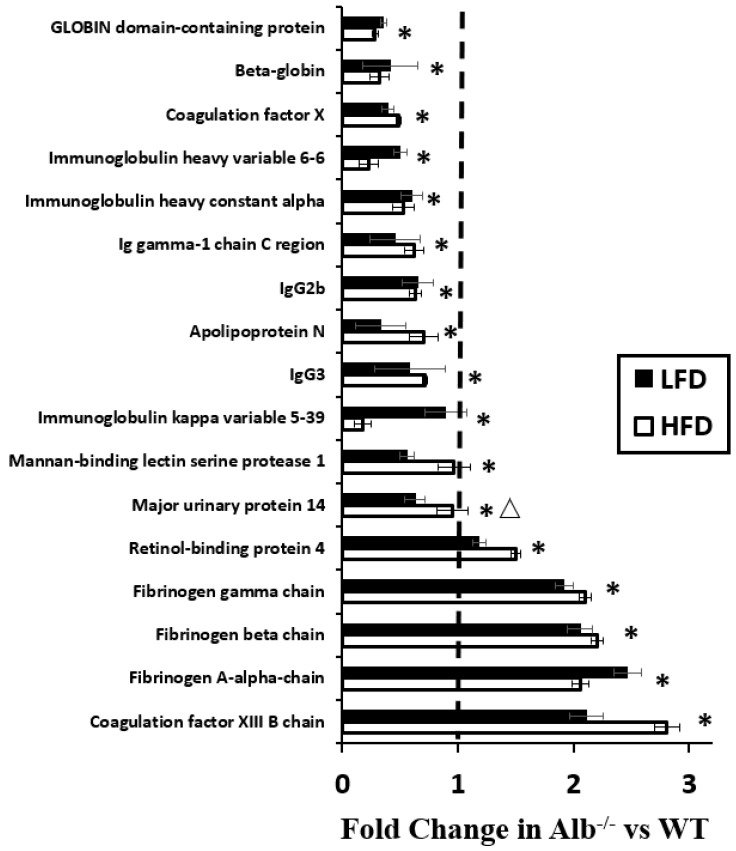
Plasma proteome. Proteins listed in the figure exhibited at least a 1.5-fold difference between WT and Alb^−/−^ mice. *n* = 5–6 mice per group. By ANOVA, each of the proteins listed in the figure achieved a significant main effect of genotype (*p* < 0.05); major urinary protein 14 exhibited a genotype-by-diet interaction. * significant main effect of genotype. ^Δ^ significant diet-by-genotype interaction. Fold change > 1 indicates Alb^−/−^ is higher than WT. Fold-change of 0.67 is equivalent to a 1.5-fold change for proteins that exhibited reduced expression in Alb^−/−^. LFD, low-fat diet; HFD, high-fat diet. Values are means ± S.E.

The global proteomics analysis was performed on plasma (Figure 9) to evaluate proteome remodeling in albumin deficiency. As expected, the detected level of albumin was nearly zero in Alb^−/−^ mice (approximately 20,000 times lower than WT. The proteins listed in Figure 9 were significantly different between WT and Alb^−/−^ mice. There were no significant main effects of diet. Overall, the magnitude of changes in plasma protein expression were fairly minor. 

## 4. Discussion

As hepatic lipid deposition is accentuated in obesity and can lead to insulin resistance, preventing lipid transfer to the liver could be beneficial. Serum albumin may facilitate aspects of metabolic dysregulation in obesity because of its involvement in inter-organ FFA transport. Lowering the export of FFA from adipose tissues could reduce the storage of lipids in other tissues including the liver, and we had hypothesized that impeding FFA–albumin binding through albumin deficiency would decrease lipid accumulation in the liver and improve insulin sensitivity markers in obese mice. Even when mice were obese and consumed high amounts of fat, albumin deficiency still led to lower plasma FFA levels, decreased hepatic steatosis, and improved glucoregulation. Furthermore, this reduction in plasma FFA concentration was achieved without exacerbation of the body fat level. Finally, Alb^−/−^ mice exhibited adaptations in liver inflammation as well as gene expression in the liver and adipose tissues that may have enhanced the favorable metabolic responses. The potential mechanisms for the observations and their implications for metabolic diseases are discussed below. 

Albumin deficiency in either obese or lean mice led to lower blood glucose levels during the OGTT and ITT, suggesting that insulin sensitivity was higher in Alb^−/−^ mice than in WT mice. Remarkably, the glucose curve during the OGTT and ITT in Alb^−/−^ mice on HFD was as low or lower than WT mice on an LFD. Therefore, albumin deficiency prevented any meaningful dysregulation of glucose metabolism when mice were challenged with obesity. As hepatic lipids and inflammation are believed to play central roles in insulin resistance, we studied lipids, inflammation, and related gene expression levels in the liver. In agreement with previous observations that steatosis is associated with insulin resistance and T2D risk [4,15], we report here that prevention of liver steatosis by albumin deficiency improved the regulation of blood glucose levels. The impact of albumin deficiency upon hepatic steatosis could be related to the reduced plasma FFA concentration, leading to a lower accumulation of both TAGs and CEs, the main storage forms of fatty acids. Lower TAG and CE accumulation may be related to reduced FFA uptake, and ORO staining confirmed the lower lipid accumulation in the liver. As the HFD that we used leads to steatosis but not advanced NASH, the elevation of liver enzymes in WT mice on the HFD was only modest as expected (not apparent based on AST levels, and elevated only moderately based on ALT levels). While there was a non-significant trend for some reduction in plasma ALT and AST levels in Alb^−/−^ compared to WT mice, albumin deficiency did not fully normalize the modestly elevated liver enzyme levels. Thus, albumin deficiency did not fully prevent the HFD-induced derangement of all liver health indicators, but based on histology and lipid analyses of liver tissues, it is apparent that albumin deficiency did protect from a substantial proportion of the liver’s obesity-related comorbidities. It is possible that ALT and AST would become more sensitive indicators of group differences in a model of more severe liver injury, such as a methionine–choline deficient diet or long-term consumption of high fat and high sugar. In the present study, hepatic lipid accumulation remains as the primary indicator of NAFLD severity. While decreased TAG accumulation likely improves insulin resistance [30], the exact mechanism is not fully elucidated. However, our results for ceramide and inflammation levels in the liver may be mechanistically relevant. There was a trend for a lower liver ceramide concentration in Alb^−/−^ mice compared to WT with the difference reaching statistical significance for one of the predominant ceramide species. This may have protected Alb^−/−^ mice from insulin resistance. Another potential connection between hepatic steatosis and insulin resistance may be inflammation. It has been shown that fatty acids, especially saturated fatty acids, can induce insulin resistance through NF-κB activation [31] via mechanisms that include signaling through Toll-like receptors [32,33]. An indicator of NF-κB activation (phosphorylated p65) was extremely low in Alb^−/−^ livers in the present study, suggesting that liver inflammation may be blunted by the absence of albumin (and likely by the reduction in plasma FFAs). It may be that low inflammation in the livers of Alb^−/−^ mice improved insulin sensitivity. In agreement with this hypothesis, it has been shown by others that KO of p65 in the liver [34] or blunted activation of NF-κB [35,36,37,38] improves insulin sensitivity. While we did not observe increased inflammation in the liver on a high-fat diet based on p65 phosphorylation, TNF-α expression, or IL-1β expression, this is likely because 8 weeks on a HFD primarily leads to an NAFLD phenotype rather than progression to non-alcoholic steatohepatitis. Nonetheless, it is noteworthy that TNF-α and IL-1β expression in the liver were similar to WT levels, and furthermore that p65 phosphorylation was very low in Alb^−/−^ mice. 

Alb^−/−^ mice on HFD had significantly higher hepatic PPAR-α expression than WT, as well as higher expression of its target gene ACADL. This finding is consistent with their lower steatosis. While the lower plasma FFA concentration in Alb^−/−^ mice likely plays a major role in reducing their hepatic steatosis, it is also possible that these gene expression levels played an additional role. Work on PPPAR-α KO mice indicated an important role of this transcription factor in hepatic fatty acid oxidation and prevention of steatosis [39]. While the steatosis reduction in the liver that we observed would be considered beneficial, it was also important to confirm that no detrimental levels of steatosis occurred in the heart, as it had been reported that whole-body inhibition of lipolysis in ATGL KO mice led to severe cardiac steatosis [26]. As shown by ORO staining, albumin deficiency actually reduced cardiac steatosis, indicating that the detriments of ATGL loss in the heart are not recapitulated by the loss of albumin. The low cardiac steatosis level in Alb^−/−^ mice may be a result of their maintained capacity for shuttling fatty acids from lipid droplets to mitochondria, as the lipolytic pathway would remain intact in albumin deficiency.

The findings for body weight and percent body fat indicate that Alb^−/−^ mice exhibit a reprogrammed metabolism that allows for avoidance of excess fat gain even though FFA release from adipose tissue is likely slowed. The body weight findings are consistent with the similar food intakes and metabolic rates between Alb^−/−^ and WT mice. While the whole-body RQ was not different between Alb^−/−^ and WT, there may have been tissue-specific changes in the relative contribution of fat and carbohydrate to mitochondrial respiration. For example, in liver and adipose tissue of Alb^−/−^ mice on the HFD exhibited higher expression than WT mice for PPAR-α and ACADL, suggesting a potentially elevated capacity to oxidize fatty acids. Adipose tissue in obese Alb^−/−^ mice, compared to obese WT mice, also exhibited higher expression of genes involved in oxidative phosphorylation which would support substrate oxidation, including oxidation of fatty acids. Furthermore, adipose tissue adapts to albumin deficiency by increasing Glut4 gene expression. This response may simultaneously improve glucoregulation and increase the capacity for re-esterifying trapped fatty acids, as the glycerol-3-phosphate for re-esterification is derived primarily from glycolytic intermediates. Re-esterification of FFAs in the adipose tissue, which is a well-documented substrate cycle [40,41,42,43,44], may also be enhanced by the elevated expression of DGAT in albumin-deficient animals. Adipose tissues in albumin deficient mice may exhibit both enhanced TAG–FFA substrate cycling as well as higher β-oxidation levels. When considering the body composition, indirect calorimetry, and tissue steatosis data as a whole, it becomes clear that the lower plasma FFA concentration in Alb^−/−^ mice leads to reduced FFA delivery to ectopic lipid deposition sites. This lower FFA delivery allows for the avoidance of steatosis development in the liver, heart, and possibly other untested organs. However, the flow of FFA is sufficient to maintain fuel supply to oxidative metabolism and to thus avoid exacerbation of whole body adiposity. Thus, FFA kinetics are altered to a moderate extent to reduce ectopic lipid deposition, yet FFA flux still proceeds at a sufficient rate to allow for the necessary amount of fat transport to support β-oxidation. 

While albumin deficiency led to metabolically advantageous adaptations, it is also important to note any detrimental changes. As we discussed previously, plasma TC concentration is increased by albumin deficiency [28], although albumin-deficient humans can have a normal lifespan [45] and we have not observed any noticeable level of premature death in our studies of albumin KO mice (unpublished observation). We observed a modest shift in the ratio of HDL-C to TC in Alb^−/−^ mice which could potentially suggest elevated LDL-C in Alb^−/−^ mice. High TC and LDL-C levels exert negative health effects [46], yet the low plasma FFA concentration in Alb^−/−^ mice could be expected to have potent benefits for health [22]. The response to albumin deficiency includes some changes in the lipoprotein profile but also beneficial changes (lower plasma FFA levels, lower steatosis, improved glucoregulation). In the future, if albumin’s FFA binding capacity moves forward as a therapeutic target, it will be important to harness the major metabolic benefits that likely stem from the lower plasma FFA level while also minimizing any impacts of the interventions upon plasma cholesterol. Certainly, translating the present findings into a clinical impact would not include efforts to reduce serum albumin concentrations in patients. Instead, the favorable changes in lipid metabolism in the albumin-deficient mouse model could be potentially achieved in humans by developing inhibitors of FFA–albumin binding. In the future, it is conceivable that drugs may be developed that block or slow the binding of FFAs to albumin, while leaving albumin in circulation to perform its other functions (such as its impacts on plasma and interstitial fluid volumes). If the development of this type of pharmaceutical was achieved, then ultimately the present findings would be translated to humans without significantly altering albumin expression in patients. Presently, the major finding is that Alb^−/−^ mice, even when they are obese, exhibit drastic improvements in glucoregulation and hepatic steatosis, and this new knowledge about the regulation of metabolism is directly relevant to the current epidemics of T2D and NAFLD. 

## 5. Conclusions

In summary, our observations are supportive of the notion that reducing plasma FFA abundance can improve insulin sensitivity. Albumin deficiency limits the plasma FFA concentration, leading to improved metabolic health, even if the mice are obese and consuming high amounts of fat. However, of course albumin reduction is not a therapeutic goal, because albumin has multiple functions. The important lesson is that intracellular lipolysis or membrane FFA transporters should not be the only focus when studying the relationship between FFA trafficking and metabolic diseases. Albumin’s role in binding FFAs is clearly also critical, and interrupting it can change systemic lipid metabolism and glucoregulation in important ways. In a scientific quest to understand the regulation of lipid metabolism in health and disease, FFA binding to albumin should be considered as a central controller of metabolic health. 

## Figures and Tables

**Table 1:** Primers and probes for qPCR with Thermo Fisher catalog numbers for the assays.

Gene	Catalog Number
18S, Hs99999901_s1	4448484
F4/80, Mm00802529_m1	4331182
TNF-α, Mm00443258_m1	4331182
Glut4, Mm00436615_m1	4331182
Adiponectin, Mm00456425_m1	4331182
Dgat1, Mm00515643_m1	4331182
Dgat2, Mm00499536_m1	4331182
CS, Mm00466043_m1	4331182
ATP5A1, Mm00431960_m1	4331182
Col1α1, Mm00801666_g1	4331182
IL1-β, Mm00434228_m1	4331182
FAS, Mm01204974_m1	4331182
PPAR-α, Mm00440939_m1	4331182
Plin2, Mm00475794_m1	4331182
CPT1a, Mm01231183_m1	4331182
ACADL, Mm00599660_m1	4331182
ACOX1, Mm01246834_m1	4331182
PPAR-γ, Mm00440940_m1	4331182

Abbreviations: TNF-α (tumor necrosis factor-alpha), Glut4 (glucose transporter-4), Dgat (diacylglycerol acyltransferase), CS (citrate synthase), ATP5A1 (ATP synthase, H+ transporting, mitochondrial F1 complex, alpha subunit 1), Col1α1 (collagen type 1 alpha 1), IL1-β (interleukin 1-beta), FAS (fatty acid synthase), PPAR-α (peroxisome proliferator-activated receptor alpha), Plin2 (perilipin 2), CPT1a (carnitine palmitoyltransferase I a), ACADL (acyl-CoA dehydrogenase long chain), ACOX1 (acyl-CoA oxidase 1), PPAR-γ (peroxisome proliferator-activated receptor gamma).

**Table 2:** Animal characteristics.

	LFD		HFD	
	WT	Alb^−/−^	WT	Alb^−/−^
Food Intake (g/day)	2.84 ± 0.15	2.77 ± 0.12	2.87 ± 0.07	2.71 ± 0.05
Body Weight (g) *	31.1 ± 0.1	28.2 ± 1.1	41.6 ± 1.2	39.4 ± 2.6
Body Fat (%) *	13.1 ± 1.4	12.9 ± 1.7	35.6 ± 1.5	31.1 ± 1.5
TC (mM) *	4.7 ± 0.3	6.5 ± 0.2 ^	6.2 ± 0.2	7.5 ± 0.3 ^
HDL-C (mM) *	1.7 ± 0.2	2.0 ± 0.1	2.3 ± 0.1	2.4 ± 0.2
HDL-C/TC ratio	0.36 ± 0.02	0.31 ± 0.003 ^	0.37 ± 0.01	0.32 ± 0.01 ^
TAG (mM) *	1.4 ± 0.11	1.6 ± 0.09	1.2 ± 0.04	1.2 ± 0.05
Insulin (μg/L)	1.0 ± 0.2	0.8 ± 0.1	2.8 ± 0.4	0.9 ± 0.1 ^
ALT (U/L) *	36.8 ± 7.1	46.0 ± 11.2	76.8 ± 11.0	60.0 ± 7.2
AST (U/L)	164.0 ± 27.1	150.0 ± 21.8	183.2 ± 31.9	159.2 ± 10.2
Exercise Performance (min)	15.9 ± 0.4	17.7 ± 0.8	14.9 ± 0.9	15.1 ± 1.3

Statistical analysis using ANOVA. ^ Alb^−/−^ different from WT within a diet, *p* < 0.05. * LFD different from HFD (main effect of diet), *p* < 0.05. Exercise capacity is expressed as time to exhaustion. Values are means ± S.E. *n* = 10–11 for body weight, and *n* = 5–6 for all other parameters. LFD, low-fat diet; HFD, high-fat diet; TC, total cholesterol concentration in plasma; HDL-C, high-density lipoprotein cholesterol concentration in plasma; TAG, triacylglycerol concentration in plasma; ALT, alanine aminotransferase; AST, aspartate aminotransferase.

## Data Availability

The data sets used and/or analyzed during the current study are available from the corresponding author on reasonable request.
